# Prophylactic Norepinephrine Infusion Reduces Postoperative Complications and Hospitalization Time in Elderly Patients Undergoing Posterior Lumbar Spinal Fusion

**DOI:** 10.1155/2021/2161036

**Published:** 2021-06-03

**Authors:** Tao Liang, Jianshe Yu, Libiao Li, Yaying Xie, Fan Wu

**Affiliations:** ^1^Department of Anesthesiology, Xuanwu Hospital, Capital Medical University, Beijing 100000, China; ^2^Department of Anesthesiology, The Affiliated Hospital of Inner Mongolia Medical University, Hohhot 010050, China

## Abstract

This single-center prospective randomized controlled trial explores the effect of prophylactic norepinephrine infusion on the incidence of complications and hospitalization time in elderly patients (60-85 years old) undergoing posterior lumbar spinal fusion. In total, 129 elderly patients were randomized into two groups: a group that received norepinephrine during general anesthesia and a control group not receiving norepinephrine. The primary outcomes were in-hospital complications and 90-day postoperative complications and hospitalization time. The results show that in-hospital complications occurred in 24 of 60 patients (40%) in the control group versus 11 of 60 patients (18.3%) in the norepinephrine group (RR, 2.182; 95% CI, 1.177–4.045; *P* = 0.015). Cardiac events occurred significantly more frequently in the control than in the norepinephrine group. Total number of patients experiencing complications within 90 days postoperatively was lower in the norepinephrine (11 of 60; 18.3%) than in the control group (26 of 60; 43.3%; RR, 2.364; 95% CI, 1.288–4.339; *P* = 0.005). The median length of hospital stay was 17 days (11–27) in the control group and 15 days (10– 23) in the norepinephrine group (*P* = 0.01). The secondary outcomes were serum levels of syndecan-1, hyaluronic acid, heparan sulfate, and brain natriuretic peptide. Logistic regression analysis is used to describe the relationship between selected independent variables and in-hospital complications. Intraoperative total fluid, crystalloid, and colloid volumes were significantly higher in the control than in the norepinephrine group. The patients in the norepinephrine group had a higher MAP but a lower heart rate than those in the control group after the induction of anesthesia and intraoperatively. Syndecan-1, hyaluronic acid, and heparan sulfate serum levels showed a different course in the two groups. In conclusion, prophylactic norepinephrine infusion during posterior lumbar spinal fusion is preferable for elderly patients undergoing lumbar spinal fusion under general anesthesia. It can reduce postoperative complications and hospitalization time by reducing the injury to the vascular endothelium. This trial is registered with Clinical Trial Registration http://www.chictr.org.cn/showproj.aspx?proj=33660, identifier ChiCTR-1900021309.

## 1. Introduction

Fluid resuscitation is considered standard of the operating room (OR). Recently, more and more people were aware of the adverse effects of fluid administration [1]. Fluid challenge administration was associated with increased perfused boundary region (and presumable volume changes of the endothelial glycocalyx) [2]. The degradation of glycocalyx was strongly associated with in-hospital mortality. This finding suggests a mechanism about intravenous fluid resuscitation strategies may lead to iatrogenic endothelial injury [3]. A better understanding of the determinants of vascular permeability is a trend about fluid resuscitation, and perhaps in the future, the identification of the endothelial glycocalyx as a possible therapeutic target [4].

Pulse pressure variation (PPV) is a derivative of the arterial pulse waveform which can be integrated into most anesthesia workstations. Rathore et al. noted that both PPV and SVV can be used to predict cardiac response to fluid loading. In both responders and nonresponders, PPV has a greater association with fluid responsiveness than SVV [5]. Its accuracy and practicality have also been demonstrated in animal experiments. Endo et al. have found that both SVV and PPV directly reflected the fluid load, and the minimum threshold values for detecting fluid responsiveness were SVV≧11% and PPV≧7% in dogs [6].

Norepinephrine has its beta-1 and alpha-1 adrenergic properties; it increases the contractility of the myocardium [7]. It could also decrease HR, PPV, and SVV. PPV is a strong predictor of fluid responsiveness in critically ill patients receiving norepinephrine. At present, the research on norepinephrine is mainly concerned with resuscitation from septic and hemorrhagic shock [8]. But, there are a few relevant research studies about prophylactic administration of norepinephrine to prevent hypotension and fluid overload undergoing lumbar spinal fusion in elderly patients during general anesthesia.

Therefore, our study is aimed at investigating the effects of prophylactic norepinephrine infusion during posterior lumbar spinal fusion on the incidence of complications and hospitalization time in elderly patients of 60-85 years old.

## 2. Materials and Methods

### 2.1. Research Design

This trial was conducted at the Department of Anesthesiology of the Affiliated Hospital of Inner Mongolia Medical University, Hohhot, China, from Nov. 1, 2018, to Nov. 1, 2019. The study was approved by the Hospital's Ethics Committee (KY2018022). All experiments were conducted following the Declaration of Helsinki. Following the ethics board's approval, the trial was registered in the Chinese Clinical Trial Registry (http://www.chictr.org.cn; ChiCTR-1900021309). Its protocol adhered to the CONSORT guidelines. After enrolment and before the start of the study, the experimental procedures, the objectives of the study, possible benefits, and side effects of the study were clearly explained to all elderly patients and their verbal and written informed consent for participation in the research.

### 2.2. Patients

Inclusion criteria are as follows: (1) age 60–85 years; (2) patients with lumbar disk herniation or lumbar spondylolisthesis scheduled to undergo elective lumbar spinal fusion with posterior lumbar discectomy, pedicle screw fixation, and intertransverse fusion; (3) American Society of Anesthesiologists (ASA) grade was I-III grades; (4) body mass index (BMI) < 30 kg/m^2^; and (5) effective communication with the physician. Exclusion criteria are as follows: (1) bradycardia (heart rate < 50 bpm); (2) cardiac morbidities, atrioventricular block higher than first degree; (3) abnormal liver or renal function; (4) hyperthyroidism; and (5) vascular disease and bowel disease (e.g., ulcerative colitis, Crohn's disease, and irritable bowel syndrome). Previous studies showed that bradycardia and related bowel syndrome are the contraindications of norepinephrine; therefore, we exclude such patients in the current study.

### 2.3. Randomization and Blinding

In total, 129 patients were included in the experiment. Among them, 9 patients declined to participate in the current study and were excluded. Finally, 120 patients were randomly divided into two groups: a control group (the same dose of normal saline) (*n* = 60) and a norepinephrine group (0.060 *μ*g kg^−1^·min^−1^ norepinephrine) (*n* = 60). A statistician used an online random number generator to perform randomization. A research assistant who did not participate in the research placed patient codes into numbered sealed opaque envelopes. The envelope was opened by an anesthesiologist resident who was not involved in the trial.

The patient's group assignment was blind to all surgeons, patients, attending anesthesiologists, and nurses.

### 2.4. Interventions

All patients drank 500 ml of clear water two hours before anesthesia induction. In the holding area, an intravenous (IV) line was established with an 18-gauge IV cannula on the forearm. Ringer's lactate (RL) solution at a rate of 5 ml·kg^−1^ was infused before general anesthesia induction and then reduced to a minimal rate. The baseline mean arterial pressure (MAP) was measured two times two minutes apart in the supine position with an automated device.

After entering the operating room, every patient was performed standard monitoring with electrocardiogram, noninvasive blood pressure, heart rate, and pulse oximetry (SpO_2_). The pressure transducer was set to zero at the midaxillary level before the radial artery cannula was performed.

In our previous study (not yet published), we had proved that 0.06 *μ*g/kg/min is better than 0.03 *μ*g/kg/min and 0.09 *μ*g/kg/min in goal-directed fluid therapy. Here, we aim to investigate the effects of prophylactic norepinephrine infusion (0.06 *μ*g/kg/min) during posterior lumbar spinal fusion on the incidence of complications and hospitalization time in elderly patients. Therefore, patients in the norepinephrine group received a continuous norepinephrine infusion at a dosage of 0.060 *μ*g·kg^−1^·min^−1^. Patients in the control group received the same amount of normal saline. Afterward, general anesthesia was induced with sufentanil (2–3 *μ*g·kg^−1^ IV), lidocaine (1.5 mg·kg^−1^ IV), and etomidate (0.3 mg·kg^−1^ IV), and neuromuscular blockade was achieved with rocuronium (0.8 mg·kg^−1^ IV). After anesthesia intubation, mechanical ventilation was performed with 50% oxygen and tidal volumes of 8 ml·kg^−1^ maintaining an end-expiratory P_ET_CO_2_ at 4–4.5 kPa. The tidal volume was reduced to 8 ml·kg^−1^ of the ideal body weight. Propofol (1.5–3 mg·kg^−1^·h^−1^) and remifentanil (0.2–0.3 *μ*g·kg^−1^·min^−1^) were used for anesthesia maintenance with a bispectral index range between 50 and 60.

The continuous infusion of 8 ml/(kg·h) lactated Ringer's solution was used to maintain the intraoperative basal fluid volume of the GDFT group. PPV was used as an indicator to evaluate the fluid administration. If PPV was less than 13% during the surgery, we infused the maintenance volume of RL at a rate of 2–3 ml·kg^−1^·h^−1^. If PPV was more than 13%, we infused RL at a rate of 2–3 ml·kg^−1^·h^−1^ and hydroxyethyl starch (HES) 130/0.4 (Voluven®, Fresenius Kabi AG, Bad Homburg, Germany) at a rate of 3 ml·kg^−1^ (ideal body weight) during 3 min to test the patient's fluid response and guide the fluid therapy. If PPV was within the target range (9-13%) and MAP below the baseline value, ephedrine 5–10 mg was started, and the patient was excluded from the analysis. If PPV was within the target range and MAP > 20% above baseline, 12.5–25 mg urapidil was administered. If PPV was within the target range and blood pressure fluctuated within 20% around the baseline, we infused RL at a rate of 2–3 ml·kg^−1^·h^−1^, and norepinephrine infusion was continued until 5 min after skin closure.

Postoperative patient-controlled analgesia (PCA) was performed with sufentanil (1.5 *μ*g·kg^−1^) combined with ondansetron 8 mg. It was diluted with physiological saline to 100 ml, 5 ml of load dose, 2 ml/h of maintenance dose, and 15 min of PCIA locking time. PCA was maintained up to 48 h after surgery.

### 2.5. Data Collection

Demographic data, including American Society of Anesthesiologists grade, duration of anesthesia and surgery, age, gender, body mass index, and perioperative complications (diabetes mellitus, hypertension, chronic obstructive pulmonary disease, preoperative anemia), were recorded. MAP and HR were assessed at the following time points: at the entrance to the operation room (Ta), 15 min after anesthesia induction (Tb), 60 mins following the surgical incision (Tc), and immediately after surgery (Td). PPV was assessed at Tb, Tc, and Td. Other indicators, such as blood loss, autologous blood transfusion, urine output, and infusion volume (crystalloids and colloids), were all recorded.

### 2.6. Blood Samples

Blood samples (6 ml each) were withdrawn from a peripheral vein at the following time points: at the entrance to the operation room (T0 = Ta), one hour after skin incision (T1), at the end of surgery (T2 = Td), and 24 h (T3) and 72 h (T4) after surgery. All blood samples were centrifuged at 3000 rpm for 15 min to collect serum and were then frozen at −80°C. Enzyme-linked immunosorbent assay kits were used to measure serum concentrations of syndecan-1 (SD-1; Biokits Tech Inc. Barcelona, Spain), hyaluronic acid (Biokits Tech Inc., Beijing, China), heparan sulfate (Wuhan Xinqidi Biological Technology Co. Ltd., Wuhan, China), and brain natriuretic peptide (BNP), according to the manufacturers' instructions. All samples were analyzed to a degree of dilution so that their concentrations were within the standard curve range.

### 2.7. Outcome Measures

The primary outcomes were in-hospital complications and 90-day postoperative complications according to Clavien-Dindo Classification System and hospitalization time. The secondary outcomes were serum levels of syndecan-1, hyaluronic acid, heparan sulfate, and brain natriuretic peptide. We performed a regression analysis to assess potential correlations between perioperative factors and outcomes. All results were registered by evaluators who did not know about the program.

### 2.8. Statistical Analysis

According to the preliminary experimental results (*n* = 20), the in-hospital complication rate was 15% in the norepinephrine and 35% in the control group. This meant that a sample size of at least 55 patients in each group would be required to achieve 95 percent capacity to detect 5 percent or more differences between groups.

Therefore, 129 patients were initially recruited to compensate for potential exclusion and follow-up losses, while a minimum sample size of at least 55 patients in each group was also ensured. Among 129 patients, 9 patients declined to participate in this study and were therefore excluded from the current study.

All quantitative data were presented as the mean and standard deviation. Intergroup comparisons were made using the unpaired Student's *t*-test, and we used repeated measures one-way analysis of variance to perform intragroup comparisons. Rank data and not normally distributed quantitative variables were presented using the median (interquartile range), using the nonparametric Mann–Whitney test for intergroup comparisons. Qualitative variables were expressed as percentages and compared with the chi-squared test. We used logistic regression analysis to describe the relationship between selected independent variables and in-hospital complications.

Statistical analysis was performed using SPSS Statistics for Windows, version 17.0 (SPSS Inc, Chicago, IL, USA). A *P* value <0.05 was considered statistically significant.

### 2.9. Results

**Figure 1:**
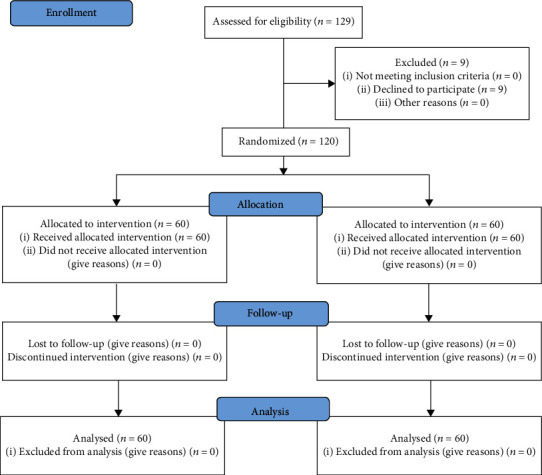
CONSORT flow diagram.

129 patients were screened for eligibility, of which nine patients were excluded, six patients did not meet inclusion criteria, and three patients quitted. Finally, 120 patients (*n* = 60) were enrolled and underwent the randomization process (Figure 1). There is no significant difference in baseline characteristics (Table 1).

The patients in the norepinephrine group had a higher MAP compared to the control group at the Tb (*P* < 0.01) and Tc time points (*P* < 0.01) but a lower heart rate (both *P* < 0.05). There was no significant difference between the two groups in the PPV at any time point (*P* > 0.05) (Table 2).

Compared with the control group, the level of SD-1 in the norepinephrine group showed a small amplitude. It rose to the highest level at the end of the operation, began to fall after 24 hours, and fell further 72 hours after the operation. However, it always remained higher than the preoperative level. BNP increased significantly in the control group at the T3 time point and fell 72 hours after the operation (*P* < 0.05) (Table 3).

The volumes of the intraoperative infusions of crystalloids (*P* < 0.01) and colloids (*P* < 0.01) and total fluid (*P* < 0.05) in the control group were considerably higher than those in the norepinephrine group (Table 4).

In-hospital complications occurred in 24 of 60 patients (40%) in the control group versus 11 of 60 patients (18.3%) in the norepinephrine group (RR, 2.182; 95% CI, 1.177–4.045; *P* = 0.015). The total number of complications was 78 in the control and 45 in the norepinephrine group. Cardiac events occurred in 26 of 60 patients (43.3%) of the control group versus 14 of 60 patients (23.3%) in the norepinephrine group (RR, 1.857; 95% CI, 1.080-3.194; *P* = 0.033) (Table 5).

### 2.10. Length of Hospital Stay, 90-Day Postoperative Complication Rate, and Mortality

The median length of hospital stay was 17 days (11–27) in the control group and 15 days (10–23) in the norepinephrine group (*P* = 0.01). The total number of patients experiencing complications within 90 days postoperatively was lower in the norepinephrine (11 of 60; 18.3%) than in the control group (26 of 60; 43.3%; RR, 2.364; 95% CI, 1.288–4.339; *P* = 0.005). According to the Clavien-Dindo classification, the majority of complications were minor (grade 1 or 2) in both groups (Table 6).

The 30- and 90-day overall mortality rates were 0 and 2.4%, respectively. No patient in the norepinephrine group, but two patients (1.67%) in the control group died (*P* = 0.156). One died of septic shock and the other of pneumonia. They both were older than 70 years.

### 2.11. Logistic Regression Analysis

We used logistic regression analysis to describe the relationship between selected independent variables (group, age, gender, BMI, surgery duration, ASAIII, BNP t3, SD-1) and in-hospital complications. We observed that there was statistical significance in following variables: group (norepinephrine vs control: OR, 0.276 (95% CI, 0.103-0.735); *P* = 0.011), age (per increasing value: OR, 1.131 (95% CI, 1.040-1.230); *P* = 0.024), ASAIII (yes vs. no: OR, 4.454 (95% CI, 1.092-11.162); *P* = 0.037), BNP (T3) (per increasing value: OR, 0.835 (95% CI, 0.414-1.233); *P* = 0.021), and SD-1 (T3) (per increasing value: OR, 1.311 (95% CI, 1.102-1.744); *P* = 0.009) (Table 7).

## 3. Discussion

This prospective randomized controlled trial showed that prophylactic norepinephrine infusion was strongly associated with reduced complication rates of in-hospital and 90-day and a shorter hospitalization time after lumbar spinal fusion in elderly patients. The rate of cardiac events was higher in the control group than in the norepinephrine group. However, there was a transient increase in serum BNP in both groups. There was no difference between the two groups in the frequency of severe cardiac complications. As we know, serum BNP is proportional to the expansion of ventricular volume. A higher BNP ratio was associated with higher subsequent all-cause and CV mortality and hospitalizations; however, the baseline BNP ratio was not associated with these outcomes. A greater decrease in BNP ratio between discharge and 30 days is associated with better outcomes [9]. Our results show that prophylactic norepinephrine administration was safe for cardiac function.

Norepinephrine acts predominantly as a vasoconstrictor and has a role in resuscitation when blood pressure cannot be maintained by fluid and blood product administration alone. In addition to its arterial vasoconstrictor effect, NE induces venoconstriction, particularly at the level of the splanchnic circulation, which increases the pressure in capacitance vessels and actively shifts splanchnic blood volume to the systemic circulation. This redistribution of the splanchnic blood volume towards the systemic circulation allows reduced intravascular volume replacement, which can be useful in a restrictive volume replacement strategy to maintain organ perfusion and avoid the effects of aggressive vascular filling [10]. Norepinephrine is a vasopressor but did not lead to some renal complications [11]. Research suggests that the intraoperative urinary output is dependent on fluid strategy but is related to the intraoperative organ perfusion pressure and the content of stress hormones [12]. However, there may be some concerns about the administration of norepinephrine through peripheral veins. A previous study suggests that 5 *μ*g of norepinephrine administered as a bolus in a peripheral venous line could treat general anesthesia-induced hypotension, with little arterial compliance when compared to PE. Furthermore, in our study, the infusion of norepinephrine we used was slow, to minimize the risk of local tissue injury [13].

Crystalloid overload and iatrogenic deterioration of the vascular permeability barrier can lead to fluid and protein transferring to the interstitium [14]. Marjanovic et al. clearly demonstrated that in the early postoperative period, intraoperative fluid overload obviously influences the anastomotic stability of the small bowel [15, 16]. Furthermore, another study showed that using a reasonable fluid strategy may improve outcome in critically ill patients. [17].

In this study, we choose the following blood parameters to be assessed: syndecan-1, hyaluronic acid, heparin sulfate, and BNP. The polysaccharide coating on the luminal surface of the vascular endothelium is a layer of glue-like substance [18]. The endothelial glycocalyx is a substance of significant function, which participates in many physiological processes; the most important is to regulate vascular permeability, prevent white blood cells and platelets from adhering to blood vessel walls, transmit shear stress, and regulate inflammation and hemostasis [19]. Hypovolemia leads to degradation of the endothelial glycocalyx, which is related to altered vascular permeability [20]. Glycocalyx degradation is accompanied by the release of its soluble components into the bloodstream-syndecan-1 and hyaluronic acid. This process is actively mediated by cleavage enzymes, including heparanase and hyaluronidase [21]. Syndecan-1 is a member of the syndecan family that comprises heparan sulfate proteoglycans. In adult human tissues, syndecan-1 is predominantly expressed in epithelial cells and plasmacytes [22]. Increases in the serum concentrations of hyaluronic acid and heparin sulfate are markers of polysaccharide coating damage. A polysaccharide coating injury may be the initiating factor of endothelial cell dysfunction and vascular access [23]. Moreover, BNP is a hormone primarily produced in the left cardiac ventricle in response to cardiac wall stretch that causes diuresis through direct natriuretic action, increased cardiac output, or decreased aldosterone levels. Elevated BNP concentrations are related to the occurrence of severe hypovolemia and possibly to hyponatremia. BNP is also a cardiac hormone produced in the heart and an established biochemical marker for heart failure (HF) because the level in plasma increases in proportion to disease severity [24].

In this study, complications were graded according to the modified Clavien-Dindo classification [25], which indicates that the rate in the control group is significantly higher than in the norepinephrine group.

There are some possible limitations in our study: one is the lack of continuous ScvO2 monitoring. Generally, a ScvO2 value of less than 70% is considered as an early indicator of tissue hypoperfusion, which is associated with an increase in postoperative complications [26, 27]. Moreover, this study is a single-center design; the sample size is relatively small. Therefore, further research and analysis are required to validate our results. Besides, it should be noted that the subjects of this study are elderly patients, the time of preoperative preparation and postoperative recovery is longer, and the related examinations before and after surgery are more. Therefore, comparing with other studies, the length of hospital stay is longer (17 days in the control group and 15 days in the norepinephrine group).

## 4. Conclusion

Prophylactic norepinephrine infusion during posterior lumbar spinal fusion is preferable for the elderly patients undergoing lumbar spinal fusion under general anesthesia. It can reduce postoperative complications and hospitalization time by reducing the injury to the vascular endothelium.

## Figures and Tables

**Table 1:** Baseline characteristics of 120 patients undergoing lumbar fusion surgery.

	Control group (*n* = 60)	Norepinephrine group (*n* = 60)	*P* value
Age, years	69.65 ± 5.83	68.48 ± 6.08	0.286
Gender, female/*n*	17	20	0.693
BMI, kg/m^2^	22.63 ± 1.01	22.40 ± 0.59	0.124
ASA level II	35	32	0.713
ASA level III	25	28	0.713
Diabetes mellitus	6	4	0.743
Hypertension	31	26	0.465
COPD	10	13	0.814
Preoperative anemia	4	6	0.743
Anesthesia duration, min	93.90 ± 5.75	91.48 ± 8.19	0.064
Operative time, min	86.71 ± 7.50	87.25 ± 8.14	0.710

BMI: body mass index; ASA: American Society of Anesthesiologists; COPD: chronic obstructive pulmonary disease.

**Table 2:** Intraoperative hemodynamics in 120 patients undergoing spinal fusion surgery.

		Control group (*n* = 60)	Norepinephrine group (*n* = 60)	*P* value
MAP	Ta	98.65 ± 3.51	97.50 ± 3.51	0.123
	Tb	67.77 ± 3.78	74.16 ± 3.99	0.000
	Tc	62.13 ± 2.70	63.87 ± 4.84	0.011
	Td	78.90 ± 4.12	79.97 ± 4.33	0.182
HR	Ta	72.75 ± 4.74	73.08 ± 4.59	0.876
	Tb	73.21 ± 3.36	62.97 ± 2.56	0.000
	Tc	71.43 ± 5.16	66.83 ± 3.67	0.000
	Td	73.36 ± 5.35	71.5 ± 4.70	0.053
PPV	Tb	12.60 ± 1.14	12.38 ± 1.11	0.292
	Tc	11.60 ± 1.14	11.43 ± 1.20	0.436
	Td	7.88 ± 1.38	7.48 ± 1.37	0.114

MAP: mean arterial pressure; HR: heart rate; PPV: pulse pressure variation.

**Table 3:** Inflammatory and cardiac biomarkers in 120 patients undergoing spinal fusion surgery.

	Time point	Control group (*n* = 60)	Norepinephrine group (*n* = 60)	*P* value
Syndecan-1 (ng/ml)	T0	9.06 ± 2.04	8.53 ± 1.03	0.073
	T1	14.51 ± 1.74	13.23 ± 1.49	0.000
	T2	17.05 ± 1.85	14.65 ± 2.19	0.000
	T3	14.98 ± 1.86	14.13 ± 1.95	0.016
	T4	14.36 ± 1.92	12.60 ± 1.59	0.114
Hyaluronic acid (ng/ml)	T0	106.00 ± 7.08	108.18 ± 8.14	0.127
	T1	129.40 ± 8.16	130.55 ± 10.19	0.496
	T2	205.33 ± 13.23	192.31 ± 9.66	0.000
	T3	190.91 ± 9.45	163.63 ± 7.98	0.000
	T4	120.15 ± 7.70	106.91 ± 7.40	0.000
Heparin sulfate (pg/ml)	T0	2074.23 ± 161.07	2092.25 ± 139.43	0.514
	T1	3157.70 ± 228.88	2947.85 ± 245.66	0.000
	T2	3852.16 ± 276.12	3649.98 ± 319.12	0.000
	T3	3249.18 ± 209.66	2667.53 ± 183.24	0.000
	T4	2768.50 ± 223.34	2673.91 ± 199.97	0.016
Brain natriuretic peptide (pg/ml)	T0	55.03 ± 6.35	54.70 ± 5.61	0.761
	T1	53.12 ± 7.53	53.25 ± 7.23	0.921
	T2	58.07 ± 7.95	59.20 ± 7.02	0.410
	T3	101.53 ± 14.34	83.50 ± 7.66	0.000
	T4	55.05 ± 6.72	56.15 ± 6.10	0.350

**Table 4:** Postoperative fluid administration in 120 patients after spinal fusion surgery.

	Control group (*n* = 60)	Norepinephrine group (*n* = 60)	*P* value
Total fluid infusion (ml)	1526.61 ± 128.59	1474.53 ± 127.30	0.028
Crystalloids (ml)	984.63 ± 113.80	846.60 ± 97.12	0.000
Colloids (ml)	599.27 ± 128.07	457.75 ± 109.97	0.000
Urine output (ml)	692.12 ± 129.30	650.31 ± 139.69	0.092
Blood loss (ml)	228.27 ± 63.73	252..2 ± 71.57	0.057
Autologous blood transfusion (ml)	105.45 ± 25.42	104.91 ± 25.44	0.909

**Table 5:** In-hospital complications in 120 patients after lumbar fusion surgery.

Category	Complication	Control group (*n* = 60); *n* (%)	Norepinephrine group (*n* = 60); *n* (%)	RR	95% CI	*P* value
Cardiac		26 (43.3)	14 (23.3)	1.857	1.080–3.194	0.033
	Acute myocardial infarction (all NSTEMI)	3	1			
	Arrhythmia	2	2			
	Congestive heart failure	3	1			
	Transient BNP increase	18	10			
Thromboembolic	Pulmonary embolism	3 (5)	1 (1.7)	3	0.321–28.031	0.619
Neurological		8 (13.3)	4 (6.7)	2	0.636–6.289	0.362
	Peripheral neuropathy	2	1			
	Delirium/agitation	6	3			
Infectious		9 (15)	6 (10)	1.5	0.569–3.953	0.582
	Urinary tract infection	2	2			
	Wound infection	4	2			
	Urosepsis	0	0			
	Pyelonephritis	3	2			
Genitourinary		3 (5)	1 (1.7)	3	0.321–28.031	0.619
	Renal failure	1	0			
	Transient increase of creatinine	2	1			
Pulmonary		17 (28.3)	9 (15)	1.889	0.915–3.898	0.12
	Pneumonia	5	2			
	Pulmonary atelectasis	6	4			
	Respiratory failure	6	3			
Blood loss	Anemia requiring postoperative transfusion	12 (20)	10 (16.7)	1.2	0.562–2.564	0.814
Complications		78	45			
Patients with complications		24 (40)	11 (18.3)	2.182	1.177–4.045	0.015
Patients with more than one complication		16 (26.7)	7 (11.7)	2.286	1.014–5.153	0.062

NSTEMI: non-ST elevation myocardial infarction; BNP: brain natriuretic peptide.

**Table 6:** 90-day postoperative complications according to Clavien-Dindo Classification System experienced by 120 patients undergoing lumbar fusion surgery.

Complication	Control group (*n* = 60); *n* (%)	Norepinephrine group (*n* = 60); *n* (%)	RR	95% CI	*P* value
Grade I	15	8			
Grade II	6	2			
Grade III	2	1			
Grade IV	1	0			
Grade V	2	0			
Total	26 (43.3)	11 (18.3)	2.364	1.288–4.339	0.005

**Table 7:** OR of selected independent variables with in-hospital complications in 120 patients undergoing lumbar fusion surgery.

Variables	OR	95% CI	*P* value
Group (norepinephrine vs. control)	0.276	0.103-0.735	0.011
Age (per increasing value)	1.131	1.040-1.230	0.024
Gender (male vs. female)	1.590	0.400-6.327	0.510
BMI (per increasing value)	1.462	0.785-2.723	0.232
Surgery duration (per increasing value)	0.980	0.918-1.046	0.545
ASA III (yes vs. no)	4.454	1.092-11.162	0.037
BNP t3 (per increasing value)	0.835	0.414-1.233	0.021
Syndecan-1 (per increasing value)	1.311	1.102-1.744	0.009

OR: odds ratio; BMI: body mass index; ASA: American Society of Anesthesiologists; BNP: brain natriuretic peptide.

## Data Availability

Raw data were generated at the affiliated hospital of the Inner Mongolia Medical University. All data included in this study are available upon request by contact with the corresponding author.
